# Regional lymph node recurrence without intragastric lesions after curative endoscopic resection of early gastric cancer meeting the absolute indications of endoscopic resection: A case report

**DOI:** 10.1097/MD.0000000000029417

**Published:** 2022-05-27

**Authors:** Hyun Wook Shin, Ji Yeon Park, Han Ik Bae, Ki Bum Park, Oh Kyoung Kwon

**Affiliations:** a Department of Surgery, Kyungpook National University Chilgok Hospital, Daegu, Republic of Korea; b Department of Surgery, School of Medicine, Kyungpook National University, Daegu, Republic of Korea; c Department of Pathology, Kyungpook National University Chilgok Hospital, Daegu, Republic of Korea.

**Keywords:** gastric cancer, endoscopic submucosal dissection, lymph nodes, recurrence

## Abstract

**Rationale:**

With the increase of gastric cancer surveillance and endoscopic resection techniques, the number of endoscopic resections being performed for the treatment of early gastric cancer in East Asian countries has been increasing. Previously, endoscopic resection has been limited to only differentiated type intramucosal cancers which had a diameter ≤2.0 cm, provided there was no evidence of ulceration and lymphovascular invasion, known as absolute indications. And recently, indications for endoscopic resection have been expanded to include even more cases.

**Patient concerns:**

A 57-year-old female, who had undergone curative endoscopic submucosal dissection for early gastric cancer under the absolute indications for endoscopic resection 5 years prior, was referred to the department of general surgery with metastatic perigastric lymph nodes without intragastric lesions.

**Diagnosis:**

Computed tomography scan revealed the presence of a few enlarged lymph nodes at the distal part of the lesser curvature of the stomach. And positron emission tomography scan further revealed the presence of two hypermetabolic lymph nodes near the common hepatic artery, suggestive of metastatic lymph nodes.

**Interventions:**

Laparoscopic distal gastrectomy and Roux-en-Y gastrojejunostomy with D2 lymph node dissection were performed.

**Outcomes:**

Final pathology report revealed the absence of any residual carcinoma in the stomach. However lymphovascular invasion of omental fat, and 3 out of 29 perigastric lymph nodes harvested had metastatic adenocarcinoma.

**Lessons:**

The case demonstrates that regional lymph node recurrence without intragastric lesions after curative resection of early gastric cancer meeting the absolute indications for endoscopic resection is possible even 5 years after resection of the primary lesion.

## 1 Introduction

Endoscopic resection is becoming prominent as the standard treatment for early gastric cancer. It is associated with a negligible risk of lymph node metastases. Furthermore, several studies have reported it to be safe and minimally invasive.^[1–3]^ The recurrence rate of early gastric cancer, following curative endoscopic resection, ranges from 4.8% to 6.1%; with metachronous recurrence being the most common form of recurrence, followed by local recurrence at the primary resection site. Extragastric recurrence is reported very rarely.^[4,5]^ Extragastric recurrence in the absence of intragastric lesions has been reported in the past for cases that met the criteria of the expanded indications of endoscopic resection.^[6,7]^

In this case report, we present a case of extragastric regional lymph node recurrence without intragastric lesions in a patient who had undergone a curative endoscopic resection, having met the criteria of the absolute indications of endoscopic resection.

Informed written consent was obtained from the patient for the publication of this case.

## 2 Case report

A 57-year-old woman was referred from the department of gastroenterology to the department of general surgery, with enlarged perigastric lymph nodes. The patient had previously undergone an endoscopic submucosal dissection (ESD) for gastric adenocarcinoma, and she underwent regular follow-up with abdominal computed tomography (CT) and esophagogastroduodenoscopy (EGD).

**Figure 1:**
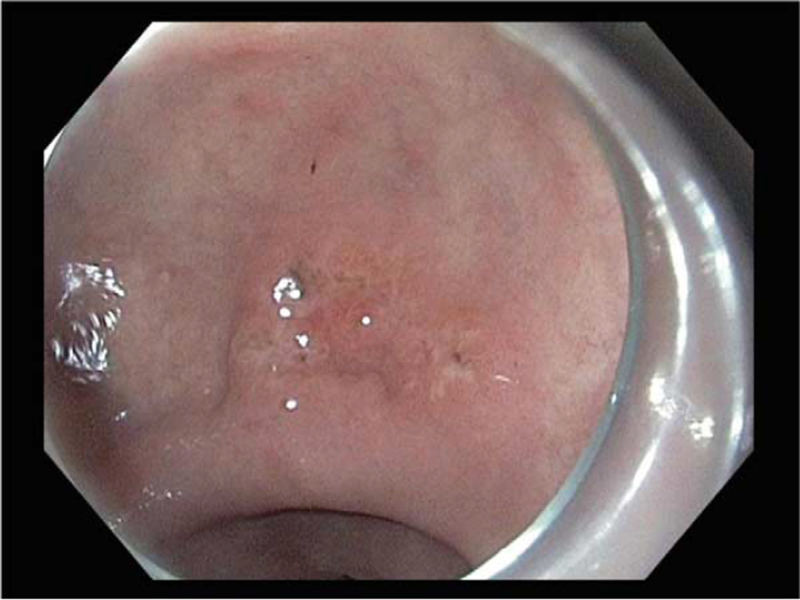
Original cancer lesion, as observed under esophagogastroduodenoscopy.

In September 2016, the patient visited the gastroenterology department seeking evaluation of an incidentally detected gastric cancer during her bi-annual nationwide screening program. The tumor was diagnosed as early gastric cancer type IIa + IIc located at the lesser curvature of the antrum (Fig. 1). The initial biopsy results suggested a diagnosis of moderately differentiated tubular adenocarcinoma. ESD was performed, and the resected tumor was 2.0 × 1.6 cm in size. The histologic type of the tumor was confirmed as moderately differentiated tubular adenocarcinoma. The depth of invasion of the lesion extended up to the muscularis mucosa, and the resection margins were free from the carcinoma. The proximal, distal, anterior, and posterior resection margins achieved by the ESD were 3, 4, 6, and 4 mm respectively. There was no evidence of either lymphatic or vascular invasion. Thus, curative ESD was performed in accordance with the guidelines of the absolute indications for endoscopic resection.^[1]^

**Figure 2:**
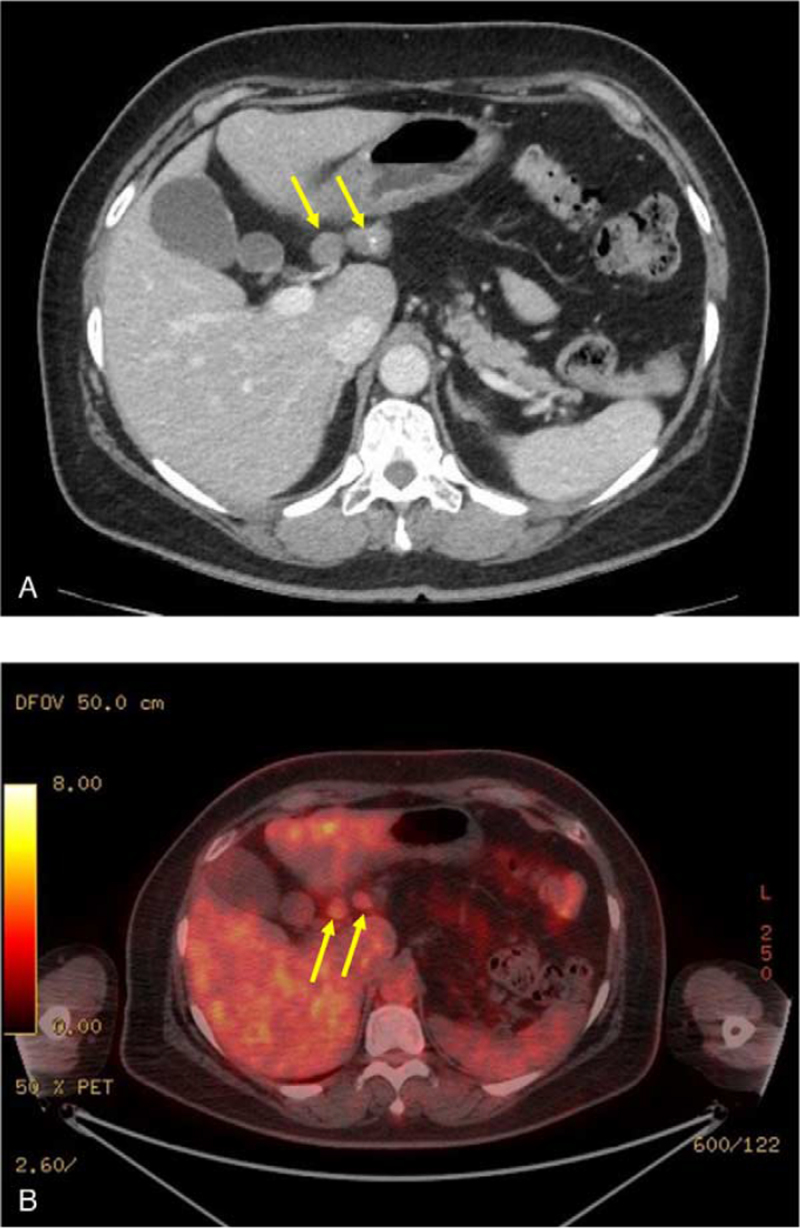
Lymph node recurrence, as observed in July 2021. (A) Computed tomography image showing two enlarged lymph nodes of size 15 and 20 mm. (B) Positron emission tomography scan image showing two hypermetabolic lymph nodes.

Yearly follow-ups were conducted with EGD and CT scans. In July 2021, on the fifth year of follow-up, there were no significant findings on EGD and two biopsies performed both displayed chronic gastritis. However, a CT scan revealed the presence of a few enlarged lymph nodes at the distal part of the lesser curvature of the stomach (Fig. 2A). Positron emission tomography (PET) scan further revealed the presence of two hypermetabolic lymph nodes near the common hepatic artery, which were suggestive of metastatic lymph nodes (Fig. 2B). There were no other findings noted on the CT scan or the PET scan which would suggest a recurrence of or metastasis from the previous gastric cancer.

Laparoscopic distal gastrectomy and Roux-en-Y gastrojejunostomy with D2 lymph node dissection were performed with a curative intent. Frozen biopsy of the enlarged lymph node at the right gastric artery (lymph node station number 5), was sent to pathology during surgery and was confirmed to be metastatic adenocarcinoma. The final pathology report revealed the absence of any residual carcinoma in the stomach. However, lymphovascular invasion of omental fat was noted, and 3 out of 29 perigastric lymph nodes harvested, had metastatic adenocarcinoma. These metastatic lymph nodes were located along the hepaticoduodenal ligament (lymph node station numbers 5 and 12a), in close proximity to the location of the original tumor.

**Figure 3:**
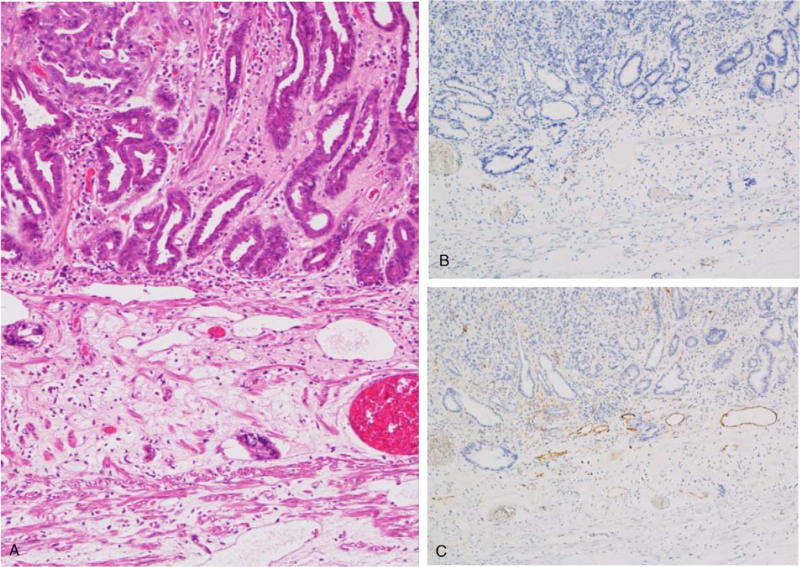
Pathology slides of the endoscopic submucosal dissection specimen. (A) Moderately differentiated adenocarcinoma invading into muscularis mucosa. (B) Immunohistochemistry stain of CD31 (vascular endothelial cells) and (C) D2-40 (lymphatic endothelial cells) revealing no lymphovascular invasion.

Examination of histopathological slides prepared from the original ESD specimen showed no evidence of lymphovascular invasion on hematoxylin and eosin (H&E) staining (Fig. 3A). Additional CD31 and D2-40 antibody staining were performed to eliminate the possibility of lymphovascular invasion undetected on H&E staining. However, neither showed any evidence of lymphovascular invasion (Fig. 3B, C).

The patient was discharged from the hospital on the fifth postoperative day and referred to the oncology department for adjuvant chemotherapy.

## 3 Discussion

With the increase of gastric cancer surveillance and endoscopic resection techniques, the number of endoscopic resections being performed for the treatment of early gastric cancer in East Asian countries, including Korea and Japan, has been increasing. Most of the studies conducted on the effectiveness of endoscopic resection for early gastric cancer have yielded favorable results, even under the expanded indications. Previously, endoscopic resection has been limited to only differentiated type intramucosal cancers which had a diameter ≤2.0 cm, provided that there was no evidence of ulceration and lymphovascular invasion, known as absolute indications. Recently, the indications for endoscopic resection have been expanded to include differentiated type intramucosal cancer with a size ≤3.0 cm in diameter with ulceration, differentiated type intramucosal cancer with a size >2.0 cm without ulceration, and undifferentiated type intramucosal cancer with a size ≤2.0 cm without ulceration, known as the expanded indications for endoscopic resection.^[1,8]^ The reason behind this expanded indication was that the possibility of lymph node metastases being present at the time of endoscopic resection was negligible.

The prevalence of lymph node metastases under the absolute and the expanded indications has been studied by several researchers previously. Oh et al^[9]^ retrospectively analyzed 1003 patients who underwent gastrectomy for early gastric cancer and reported that 0 out of 204 (0%) patients who satisfied the absolute indications and 6 out of 700 (0.9%) patients who satisfied the expanded indications had lymph node metastases. A meta-analysis that included 12 studies and comprised a total of 9798 patients also reported that 6 out of 3025 (0.2%) patients who satisfied the absolute indications, and 68 out of 9798 (0.7%) patients who met the expanded indications, had lymph node metastases.^[10]^ Of the 12 studies in the meta-analysis, 9 reported no lymph node metastases in patients who met the absolute indications. Moreover, of the 3 studies that reported lymph node metastases in patients who met the absolute indications,^[11–13]^ the study by Choi et al^[13]^ did not exclude lymphovascular invasion from the absolute indications. Therefore, the 3 cases of lymph node metastases reported by Choi et al,^[13]^ which account for half of the number of cases of lymph node metastases meeting the absolute indications in the meta-analysis, might not have been included if lymphovascular invasion was accounted for. Consequently, it can be assumed that the incidence of lymph node metastases meeting the absolute indications for endoscopic resection was even lower than 0.2%.

We encountered a case that met the absolute indications for endoscopic resection and was subsequently diagnosed with lymph node metastases in the fifth year of follow-up, without recurrence in the remnant tissue of the stomach. Since there was no evidence of tumor in the remnant of the stomach, it was assumed that the lymph node metastases originated from the primary cancer 5 years prior. A possible explanation is that there was unobserved lymphovascular invasion at the time of endoscopic resection. A thorough review of the original ESD specimen using antibody staining was conducted for this reason. However, no evidence of lymphovascular invasion was found. It is also noteworthy that a period of 5 years had passed since the presentation of the primary cancer before the lymph nodes became sufficiently enlarged to be detected by CT. This can be explained with the hypothesis that the isolated residual cancer cells can remain dormant for years after primary treatment and then re-proliferate.^[14]^ In this case isolated residual cancer cells that resided in the lymph node at the time of ESD may have gone through a period of dormancy and re-proliferated few years after the procedure.

Extragastric recurrence after curative endoscopic resection is extremely rare. In a study that evaluated 4015 patients who underwent endoscopic resection, only 15 patients had extragastric recurrence.^[6]^ Extragastric recurrence without intragastric lesions occurred in 9 patients. However, 6 of them had not undergone curative resection, and the remaining 3 patients underwent the procedure under the expanded indications for endoscopic resection. Only one case of extragastric recurrence was reported that underwent curative resection under the absolute indications for endoscopic resection. However, this case of extragastric recurrence was accompanied by the recurrence of intragastric advanced gastric cancer. The authors concluded that extragastric recurrence is always accompanied by intragastric recurrence in patients who meet the absolute indications. However, this conclusion is contradictory to the findings in our case.

There have been no reported instances of regional lymph node recurrence in the absence of intragastric lesions after curative endoscopic resection in patients meeting the absolute indications, such as the case in this report. However, the case with the closest similarity was that by Kamiya et al,^[7]^ which presented with extragastric recurrence without intragastric lesions, after curative endoscopic resection of well-differentiated intramucosal early gastric cancer having a size of 30 mm without evidence of ulceration and lymphovascular invasion. The findings of this case are consistent with the findings of Lee et al^[6]^ who reported 3 cases of regional lymph node recurrence without intragastric lesions from among 1302 patients who underwent curative resection under the expanded indications for endoscopic resection. Considering the possibility of extragastric recurrence without intragastric lesions within the patient-group included under the expanded indications, Lee et al^[6]^ also emphasized the importance of CT scan during follow-up for patients undergoing endoscopic resection under the expanded indications. However, our case presents a risk of extragastric recurrence without intragastric lesions, even in patients meeting the absolute indications. Therefore, follow-up using CT scan should also be considered for patients who have undergone curative endoscopic resection for early gastric cancer, even if the patient meets the absolute indications.

## 4 Conclusion

Regional lymph node recurrence without intragastric lesions after curative resection of early gastric cancer meeting the absolute indications for endoscopic resection is possible even 5 years after resection of the primary lesion. Careful follow-up of all patients who underwent endoscopic resection for early gastric cancer, regardless of absolute or expanded indications, should be conducted with abdominal CT and EGD for at least 5 years.

## Author contributions

**Conceptualization:** Ji Yeon Park.

**Data curation:** Hyun Wook Shin.

**Formal analysis:** Han Ik Bae.

**Investigation:** Hyun Wook Shin.

**Supervision:** Ji Yeon Park, Ki Bum Park, Oh Kyuong Kwon.

**Validation:** Han Ik Bae.

**Writing – original draft:** Hyun Wook Shin.

**Writing – review & editing:** Hyun Wook Shin, Ji Yeon Park.
